# Can We Consider Erectile Dysfunction as an Early Marker of Cardiovascular Disease?∗

**DOI:** 10.1016/j.jacadv.2023.100384

**Published:** 2023-06-30

**Authors:** Francesca Cortese, Marco Fabio Costantino, Giampaolo Luzi

**Affiliations:** a Cardiology Unit, “Madonna Delle Grazie Hospital”, Matera, Italy; b SSD Cardiovascular Imaging Department, AOR S. Carlo, Potenza, Italy; c Cardiac Surgery Unit, Department of Cardiovascular Science, San Carlo Hospital, Potenza, Italy

**Keywords:** endothelial dysfunction, subclinical cardiac dysfunction, cardiovascular risk

**Figure:**
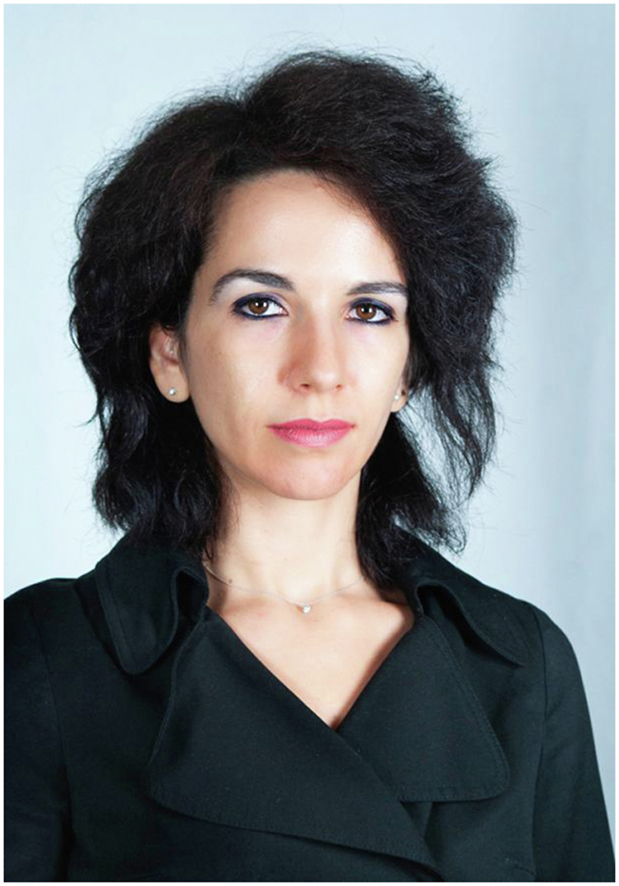

Erectile dysfunction (ED) is the inability to achieve or maintain an erection that is sufficient for satisfactory sexual performance. This problem affects a considerable proportion of men at least occasionally, and it is estimated that around 52% of men experience some form of ED with an increase from about 5 to 15 percent between ages 40 and 70 years.^1^^,^^2^ Although it is not a lethal condition, the interest surrounding ED and its remedies has been constant and present in history since the 15th century when Leonardo da Vinci described the increase in blood flow in the penis as a cause of erection.^3^ The causes of ED are classified into 2 main categories: psychogenic and organic causes, the last representing 80% of the total. Organic causes can be broadly divided into nonendocrine and endocrine. Among the nonendocrine etiologies, the vasculogenic (affecting blood supply) is the most common and can involve arterial inflow disorders and abnormalities of venous outflow (corporeal veno-occlusion); there are also neurogenic (affecting innervation and nervous function) and iatrogenic (relating to a medical or surgical treatment) etiologies. Among endocrine factors leading to ED, reduced serum testosterone levels have been implicated, but the exact mechanism has not been fully elucidated.^3^

The incidence of ED increases with age, passing from 20 to 40% in men 60 to 69 years to 50 to 100% in men 70 to 80 years, depending on the differing definitions of ED in various studies.^4^ In addition to age, cardiovascular (CV) risk factors such as hypertension, diabetes, smoking, obesity, and dyslipidemia are significantly associated with ED, and this has led over time to hypothesize a close link between ED and CV disease.^5^ The interaction between androgens, chronic inflammation, and CV risk factors can determine endothelial dysfunction and atherosclerosis, resulting in disorders of penile and coronary circulation. Evidence demonstrated that ED and vascular diseases share a similar pathogenic involvement of nitric oxide pathway leading to impairment of endothelium-dependent vasodilatation (early phase) and structural vascular abnormalities (late phase).^5^

The smaller size of penile artery size compared with coronary arteries may explain why the same level of endothelial dysfunction can cause a more significant reduction of blood flow in erectile tissues compared with that in coronary circulation.^6^ Nowadays, the correlation between ED and CV disease has been widely demonstrated, and ED often precedes CV events and can be used as an early marker to identify men at high risk of major CV disease.^7^

A recent umbrella review of systematic reviews and metanalysis showed a higher risk of CV diseases (relative risk [RR]: 1.45; 95% CI: 1.36-1.54; *P* < 0.001), coronary heart disease (RR: 1.50; 95% CI: 1.37-1.64; *P* < 0.001), CV-related mortality (RR: 1.50; 95% CI: 1.37-1.64; *P* < 0.001), all-cause mortality (RR: 1.25; 95% CI: 1.18-1.32; *P* < 0.001), myocardial infarction (RR: 1.55; 95% CI: 1.33-1.80; *P* < 0.001), and stroke (RR: 1.36; 95% CI: 1.26-1.46; *P* < 0.001) in patients with endothelial dysfunction than in other patients.^8^

The paper by Selvin et al^9^ in this issue of *JACC: Advances* conducted cross-sectional analysis using logistic regression to examine association of elevated cardiac biomarkers [N-terminal-pro BNP (NTproBNP), high sensitivity troponin T (hs troponin T) and 3 high sensitivity troponin I (hs troponin I) assays, >90th percentile], and mortality in 2,971 males with ED age 20 years or older in the National Health and Nutrition Examination Survey 2001 to 2004.

The study population included 2,971 men without a history of CV disease (self-reported coronary heart disease, angina, heart attack, stroke, or heart failure), who had valid assessments of ED and cardiac biomarkers. Hs-troponin T and NTproBNP (Roche Diagnostics) and hs-troponin I (with 3 different assays, Roche Diagnostics, Abbott Laboratories, and Siemens Healthcare Diagnostics) were measured in the study population.

ED was only assessed during the 2001 to 2004 NHANES survey cycles: all participants were asked to self-report ED during a computer-assisted self-interview. Only the subjects who answered that they were never able to get and keep an adequate erection were considered in the study.

Demographic (age, sex, education, marital status, race, and ethnicity) and anthropometric (body mass index) parameters were collected, as well as the presence of hypertension, hypercholesterolemia, and diabetes. CV mortality, defined as heart disease or cerebrovascular disease, was collected in NHANES via probabilistic linkage to the National Death Index (end of follow-up December 31, 2019).

The global prevalence of severe ED (never able to achieve an erection) was 4.5%, corresponding to 4.4 million men age 20 years or older, and was much higher among men age 65 or older (26.2%). The mean age among men with severe ED was 65 years, 23 years older than men without ED. After the correction for age, NTproBNP levels were similar in subjects with and without ED, whereas elevated hs-troponin was more common in men with ED. In the multivariable (race, education, marital status, smoking, body mass, hypertension, hypercholesterolemia, and diabetes)-adjusted model, elevations of hs-troponin T and of the 3 hs-troponin I assays were associated with ED, although the associations with elevated hs-troponin I were not statistically significant. Hs-troponin T was more strongly associated with ED as compared to the hs-troponin I. There were 673 deaths (191 due to CV causes) during a median of 16 years of follow-up. Men reporting ED were at a significantly elevated risk of all-cause (HR: 1.23; 95% CI: 1.04-1.46) and a nonsignificantly elevated risk of CV mortality (HR: 1.35; 95% CI: 0.92-1.10) after accounting for other risk factors. Men experiencing ED and elevations in any of the cardiac biomarkers were at the highest risk of subsequent mortality with a consistently >2-fold increased risk of CV death. The association between muscle disorders and frailty from one hand and elevation of hs-troponin T from the other may explain the more robust associations of hs-troponin T with ED.^10^ On the contrary, NTproBNP is a hemodynamic biomarker secreted by cardiomyocytes in response to myocardial wall stretch and has a weaker association with the vascular disease processes that underlie ED.^11^

In conclusion, men with ED had a higher prevalence of subclinical cardiac dysfunction, expressed by elevated cardiac biomarkers, and were at a markedly higher risk of CV death.

This association may determine 2 practical consequences: first the close link between endothelial dysfunction and ED showed that an adequate prevention of CV risk factors (smoking, body mass, hypertension, hypercholesterolemia, and diabetes) may prevent ED, second an early identification of ED may help to identify subjects at higher CV risk in order to apply adequate intervention strategies.

## Funding support and author disclosures

The authors have reported that they have no relationships relevant to the contents of this paper to disclose.
